# A biophysical neural network model for visual working memory that accounts for memory binding errors

**DOI:** 10.1186/1471-2202-16-S1-P8

**Published:** 2015-12-18

**Authors:** João Barbosa, Albert Compte

**Affiliations:** 1 Intitut d'Investigacions Biomèdiques August Pi i Sunyer (IDIBAPS), 08036, Barcelona, Spain

## 

Binding errors, also called swap errors, occur in working memory tasks when the participant fails to report the feature of a previously presented target but the response is accurate relative to a non-target stimulus (for instance, s/he reports the location of the green item when the red item's location was to be reported). Psychophysical experiments have shown the importance of these errors in working memory tasks [1-3], and they reflect the failure of the system to maintain bundled through memory the conjunction of features that define one object. How the brain is able to integrate and maintain integrated several features of an item in one single memory is still an open question. Conjunctive selectivity, where single units are selective to combination of stimuli, has been suggested as a possible solution [4]. However, with the increase of features to encode the number of necessary neurons escalates exponentially. A hypothesis formulated for this problem in visual perception states that synchrony of different neural assemblies coding each of them a single feature of an item plays the main role [5]. This is an attractive hypothesis, because of its parsimonious and flexible neural representation: a particular neuron can participate in the coding of different objects at different times. Because under this hypothesis there is no hardwired selectivity for each group of features, it allows ex novo representations to emerge. Current neurophysiological data provides partial support for this idea but the specific neural mechanism remains unknown [5,6].

In order to understand this phenomenon and to test this synchrony hypothesis, we built a biophysical neural network model for the storage of items orks for working memory (ring models, as in [7]), one representing colors and the other one locations of equal eccentricity. These two networks are then connected via weak cortico-cortical excitation through AMPA synapses. With this model we are able to maintain persistent activity through recurrent synaptic interactions in memory bumps [8] that encode colors and locations. AMPA synapses in recurrent excitation within each network induce gamma oscillations during bump activity through the interplay of fast excitation and slower feedback inhibition [7]. We found that when multiple bumps are maintained within one ring model, the corresponding bump attractors oscillate out of synchrony with each other. In this model, the binding between color and location is effectively accomplished through the synchronization of gamma oscillations between individual bumps in the two networks as a result of weak cortico-cortical excitation. As previously proposed [9-10], in our network model different memories are held at different phases of a low-frequency oscillation. In some simulations swap errors arise: "color bumps" abruptly change their phase and thus change their synchrony relationship with "location bumps". This leads to a wrong association between color and location that may underlie memory binding errors reported in working memory tasks.
